# Direct Electrochemistry of Hemoglobin at a Graphene Gold Nanoparticle Composite Film for Nitric Oxide Biosensing

**DOI:** 10.3390/s130607492

**Published:** 2013-06-07

**Authors:** Miao-Qing Xu, Jian-Feng Wu, Guang-Chao Zhao

**Affiliations:** College of Environmental Science and Engineering, Anhui Normal University, Wuhu 241000, China; E-Mails: mqxu2010@mail.ahnu.edu.cn (M.-Q.X.); jfengwu@mail.ahnu.edu.cn (J.-F.W.)

**Keywords:** hemoglobin, graphene, gold nanoparticles, nitric oxide, biosensor

## Abstract

A simple two-step method was employed for preparing nano-sized gold nanoparticles-graphene composite to construct a GNPs-GR-SDS modified electrode. Hemoglobin (Hb) was successfully immobilized on the surface of a basal plane graphite (BPG) electrode through a simple dropping technique. Direct electrochemistry and electrocatalysis of the hemoglobin-modified electrode was investigated. The as-prepared composites showed an obvious promotion of the direct electro-transfer between hemoglobin and the electrode. A couple of well-defined and quasi-reversible Hb CV peaks can be observed in a phosphate buffer solution (pH 7.0). The separation of anodic and cathodic peak potentials is 81 mV, indicating a fast electron transfer reaction. The experimental results also clarified that the immobilized Hb retained its biological activity for the catalysis toward NO. The biosensor showed high sensitivity and fast response upon the addition of NO, under the conditions of pH 7.0, potential -0.82 V. The time to reach the stable-state current was less than 3 s, and the linear response range of NO was 0.72-7.92 μM, with a correlation coefficient of 0.9991.

## 1. Introduction

Graphene, a single layer of sp^2^-hybridized carbon atoms packed into a dense honeycomb two-dimensional lattice, has been the subject if intensive investigations by both the experimental andtheoretical scientific communities since the experimental observation of single layers by Novoselov and Geimin in 2004 [1–3]. Due to its novel properties, such as exceptional thermal and mechanical properties, high electrical conductivity, graphene may be a promising alternative to carbon nanotubes for synthesizing nanocomposites, nanoelectronics, electromechanical reasonators, and ultrasensitive sensors [4–6]. Like carbon nanotubes (CNTs) and other nanomaterials, a key challenge in the synthesis and processing of bulk-quantity graphene sheets is aggregation due to the highly cohesive van der Waals energy (5.9 kJ·mol^−1^ carbon) [7] that adheres graphitic sheets to one another. To overcome this problem, graphene sheets have been prepared in the presence of a broad variety of reagents, including octadecylamine, polystyrene, poly (sodium 4-styrene sulfonate), alkali metal and even Au nanoparticles [8–10].

On the other hand, due to its low-cost, large specific surface area and strong interactions with metal clusters, graphene is a promising material for catalytic applications [11]. The dispersion of metal nanoparticles on graphene sheets potentially provides a new way to develop catalytic materials [12] used as electrocatalysts for oxygen reduction [13,14] and methanol oxidation [15,16]. Among noble nanoparticles, gold nanoparticles (GNPs) are one of the most studied nanomaterials, due to their remarkable properties [17]. Up to now, a number of studies have been made on the preparation of hybrid materials containing graphene and AuNPs through various functionalization strategies with potential applications in many fields such as catalysis, electronics, and biology [18–20]. For example, Luan *et al.* [21] employed a GNPs-graphene nanostructure film to construct Au/graphene/HRP/CS modified electrode and the resulting biosensor exhibited excellent electrocatalytic response to H_2_O_2_. Huang *et al.* [22] also showed that a GNPs/graphene-NH_2_ nanocomposite could effectively facilitate the direct electron transfer of catalase to the electrode.

However, to date, there are few papers concerning the electrochemical response of hemoglobin on gold nanoparticles-graphene sheets composites. As a well-characterized, electron transfer protein, hemoglobin has been used extensively as a test system for direct electron transfer (DET) of redox proteins or for communication in a protein stack by assembling it with other proteins in a matrix [23–25].

In this work, we synthesized Au nanoparticles using graphene oxide (GO) as protecting agent to give a stable red suspension. To obtain high electrical conductivity and water solubility of our GNPs-graphene composites, we added sodium dodecyl sulfate (SDS) solution to the GNPs-GO suspension prior to chemical reduction. The anionic surfactant SDS was employed as an effective solubilizing agent for graphene nanosheets and for isolating them from each other. Otherwise, the strong van der Waals interactions among the reduced graphene sheets results in their rapid aggregation in solution during the chemically reduction process. Then, water soluble GNPs-GR can be successfully obtained by the non-covalent interaction of graphene with sodium dodecylsulphate (SDS) in this procedure [26,27]. Further, we also demonstrate the direct electrochemistry and electrocatalysis of hemoglobin (Hb) immobilized in GNPs-GR-SDS films for the detection of NO.

## 2. Experimental

### 2.1. Apparatus and Reagents

Electrochemical experiments were performed with a CHI660 electrochemical analyzer (CHI, Austin, TX, USA) with a conventional three-electrode cell. A Hb/GNPs-GR-SDS modified BPGelectrode (Φ = 5.2 mm) was used as a working electrode. An Ag/AgCl electrode and a platinum wire electrode were used as the reference and the auxiliary electrodes, respectively.

Horse heart hemoglobin was obtained from Sigma (St. Louis, MO, USA) and used without purification. Hb solution (5 mg·mL^−1^) was stored at a temperature of 4 °C as stock solution. Graphite, and hydrazine hydrate solution (50 wt %) were purchased from Shanghai Chemical Reagent Co. Ltd. (Shanghai, China) Saturated NO solutions were prepared as described in the previous literature [28]. In brief, double-distilled water was bubbled with high purity nitrogen for 15 min to remove oxygen and then the water was bubbled with pure NO gas for 30 min to prepare a saturated NO solution. Other chemicals were of analytical grade and used without further purification. All solutions were made up with doubly distilled water and deaerated with high purity nitrogen before performing the experiments and maintained under nitrogen atmosphere during measurements. Scanning electron microscopy (SEM) image was obtained using a LEO1530 field emission SEM system (Leo Co., Oberkochen, Germany).

### 2.2. Synthesis of GNPs-GO Suspension

The oxidation of the graphite was carried out based on the Hummers method [29]. The obtained graphene oxide was washed with deionized water six times to remove the remaining metal ions and acid and stored for use. We prepared GNPs-GO nanosheets by chemical reduction of HAuCl_4_ with NaBH_4_ in the presence of aqueous graphene oxide (GO). An aqueous solution of HAuCl_4_ (40 μL, 58 mM) was added into graphene oxide aqueous solution (4 mL, 0.45 mg·mL^−1^) and stirred for 5 min. After that, slow and controlled addition of NaBH_4_ solution (40 μL, 0.2 M) is essential to avoid gold nanoparticles from forming too fast and aggregating. This experiment was repeated with graphene oxide concentrations of 0.02, 0.11 and 0.27 mg· mL^−1^ as well as one experiment where no graphene oxide was added.

### 2.3. Chemically Reduction of GNPs-GO Nanosheets

SDS solution (1 mL, 0.1 M) was added to the obtained gold nanoparticles-graphene oxide suspension and stirred for 20 min. Then, hydrazine hydrate solution (20 μL) was added and stirred for 5 min. This mixture was reacted at 100 °C for 24 h. The GNPs-GO solution changed color, from red to black, after the hydrazine-vapor treatment, indicating reduction of the graphene oxide [22]. The resulting black solution (GNPs-GR) can remain as a fine dispersion for at least three months.

### 2.4. Electrode Preparation

Initially, the BPG electrode was polished with abrasive paper before each experiment. After a short rinse with distilled water and ultrasonic cleaning with distilled water and ethanol, the electrode was allowed to dry at room temperature. Then, the GNPs-GR-SDS suspension (10 μL) was cast on the surface of a pretreated BPG electrode with a microsyringe and the solvent (water) was allowed to evaporate at ambient temperatures. The electrode was denoted as the GNPs-GR-SDS/BPG electrode. Finally, this GNPs-GR-SDS/BPG electrode was immersed in a phosphate buffer solution (pH 7.0) containing 5 mg·mL^−1^ Hb for 3 h to form an Hb/GNPs-GR-SDS/BPG electrode. Prior to electro-chemical experiments, the electrode was rinsed thoroughly with doubly distilled water andstored at 4 ° C. The Hb/GR-SDS modified electrode without GNPs and the Hb/GNPs-SDS modified electrode without graphene were prepared in the same way, only omitting the steps of the addition of HAuCl_4_ solution and graphene oxide solution, respectively.

## 3. Results and Discussion

### 3.1. Characterization of the GNPs-GO Composite

**Figure 1.**
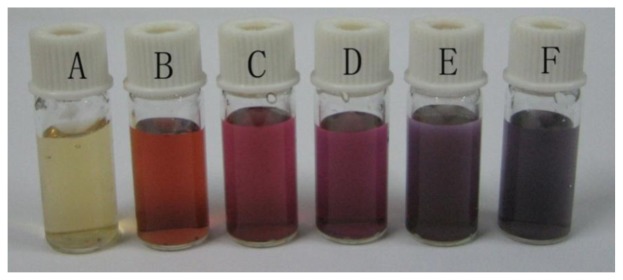
Photograph of graphene-oxide (**A**) and 0.58 mM gold nanoparticles solutions containing different concentrations of graphene-oxide (**B**–**F**). Concentration of graphene-oxide (**from A to F**): 0.45, 0.45, 0.27, 0.11, 0.02 and 0 mg·mL^−1^.

A photograph of five GNPs-GO suspensions with different graphene oxide concentrations is shown in Figure 1. These absorption changes indicate that the graphene-oxide plays an important role in dictating the size of gold nanoparticles. Individual small gold nanoparticles appear red; however, when particles aggregate together, the plasmon resonances can combine. Plasmon resonance associated-absorption wavelengths will shift from blue to red, and reflected light will shift from red to blue, which results in further color changes of the gold nanoparticle solutions. This important feature in turn enables us to modulate the surface plasmon absorption of gold-graphene composites. When 0.45 mg·mL^−1^ graphene oxide was added into the solution, the color of the solution turned a red wine color. Reducing the amount of graphene oxide gradually, the color of the solution turns red, then purple, blue, which could be explained by the aggregation of gold nanoparticles. At a low graphene concentration (0.02 mg·mL^−1^), the gold particles are suspended but in an aggregated state. Without the addition of any dispersant, the suspension flocculates very rapidly, which should indicate a strong agglomeration of the nanoparticles.

**Figure 2.**
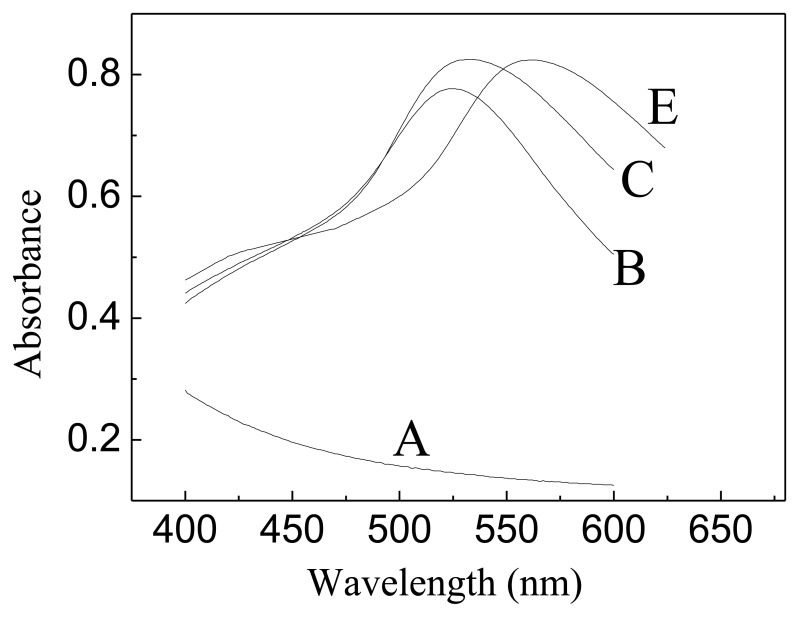
Absorption spectra of graphene-oxide (**A**) and 0.58 mM gold nanoparticles solution containing different concentrations of graphene-oxide (**B**–**E**): 0.45, 0.27 and 0.02 mg·mL^−1^.

Figure 2 shows the absorption spectra of GNPs-GO suspensions with different color. As expected, addition of less GO resulted in a small red-shift in the ë_max_ from 520 to 556 nm, which is typical for increasingly larger GNP diameters. Similar red-shifts have been observed in the measured optical spectra of gold nanoparticles and are attributed to the effect of electromagnetic retardation in larger nanoparticles [30,31].

In other words, gold nanoparticles-graphene oxide composites were successfully synthesized using graphene oxide as a dispersing agent to optimize the dispersion and the stability of the gold nanoparticles. Unlike the methods reported in previous literatures, the present method involves a facile fabrication route for the attachment of gold nanoparticles on the surface of graphene without anyorganic reagent. The resulting GNPs-GO suspension can remained as a fine dispersion at least two months. Subsequently, we added hydrazine hydrate to the GNPs-GO suspension to obtain a gold nanoparticle-graphene solution in the presence of sodium dodecyl sulfate.

**Figure 3.**
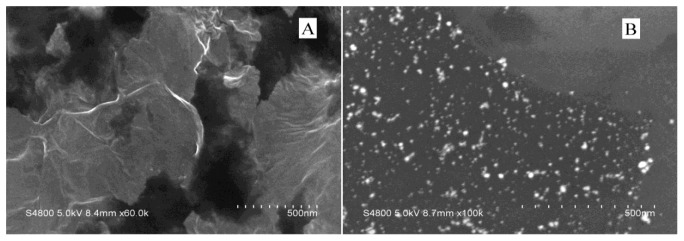
SEM images of graphene sheet (**A**) and gold nanoparticles attached on a graphene sheet (**B**).

Figure 3 depicts scanning electron micrographs (SEM) of the graphene nanosheets before and after modification of gold nanoparticles using 0.45 mg·mL^−1^ graphene. The SEM analysis indicates that the gold nanoparticles are uniformly deposited on the graphene surface, with an average size of 50 nm. This fact showed that the presence of the oxygen functionalities at the graphene surface may play an important role on the nucleation and growth of gold nanoparticles. Furthermore, Figure 3(B) also implies that the modification is not homogeneous, which may be due to non-specific binding between the gold nanoparticles and graphene oxide nanosheets. For example, Wang's group have fabricated GNPs and poly(diallyldimethylammonium chloride) (PDDA)-functionalized graphene nanocomposites through electrostatic interaction, which possess a smaller average particle size and a more mono-disperse size range [32].

### 3.2. Direct Electrochemistry of Hb on GNPs-GR-SDS/BPG Electrode

**Figure 4.**
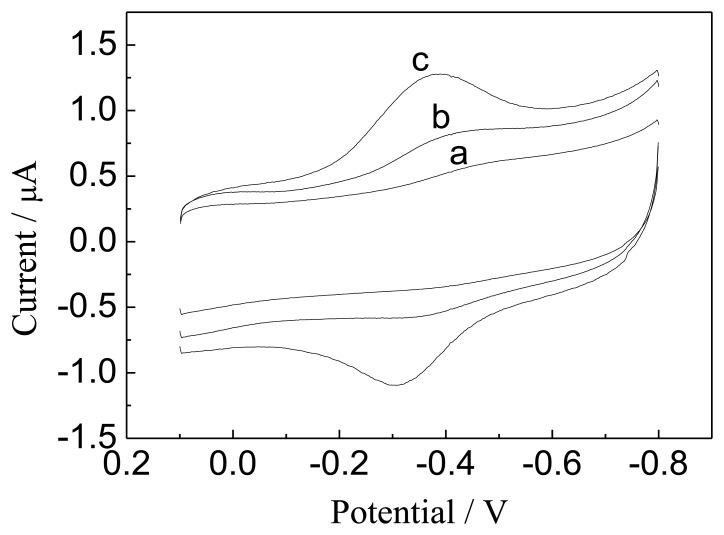
Typical CVs of (**a**) Hb/GNPs-SDS/BPG, (**b**) Hb/GR-SDS/BPG, (**c**) Hb/GNPs-GR-SDS/BPG electrode in 0.1 M PBS, pH 7.0, with a scan rate of 100 mV·s^−1^.

Figure 4 displays typical cyclic voltammograms (CVs) obtained at different Hb-modified electrodes in 0.1 M N_2_-saturated Hb-free phosphate buffer solution (pH 7.0). The Hb/GNPs-GR-SDS/BPG electrode gives a couple of stable and well-defined redox peaks, where anodic and cathodic peak potentials are located at −317 and −398 mV, respectively (curve c in Figure 4). The separation of anodic and cathodic peak potentials (ΔEp) is 81 mV, indicating a fast electron transfer reaction. The shapes of the redox peaks were symmetric and the peak currents of anode and cathode were essentially equal, suggesting that electroactive Hb moieties on the surface of the electrode are reduced during the forward negative scan and are oxidized fully again when the potential scan was reversed.

Besides, the Hb/GR-SDS modified electrode (curve b in Figure 4) also displays a couple of Hb redox peaks, however, these peak currents are much smaller than those at the Hb/GNPs-GR-SDS modified electrode (curve c in Figure 4). At the Hb/GNPs-SDS modified electrode (curve a in Figure 4), almost no redox peak could be observed. This reveals that the introduction of graphene sheets into the GNPs-GR-SDS composite strongly increased the electrocatalytic activity and stability of Hb. Furthermore, the combination of graphene and gold nanoparticles have a significant effect on the kinetics of the electrode reaction for the proteins and provide a suitable network-like microenvironment for the proteins to transfer electrons to the BPG electrode.

**Figure 5.**
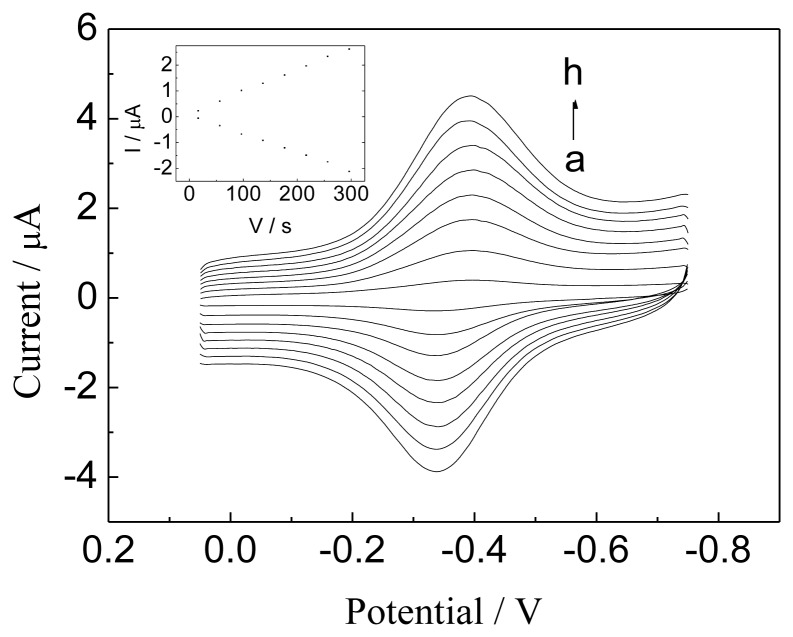
CVs of the Hb/GNPs-GR-SDS/BPG electrode in N_2_-saturated PBS (pH 7.0) at different scan rates. Scan rate (from a to h): 0.06, 0.1, 0.14, 0.18, 0.22, 0.24, and 0.3 V·s^−1^. The inset plot shows the linear dependence of anodic and cathodic peak currents vs. scan rate in the range 0.06-0.3 V·s^−1^.

Figure 5 illustrates the CVs of Hb/GNPs-GR-SDS modified electrode in different scan rates in the range 0.06–0.3 V s^−1^. The peak current linearly increased with the scan rate with a correlation coefficient of 0.995, suggesting that this is a surface-controlled process. The electron transfer rate constants (Ks) can be estimated according to Laviron [33], which is 48 ± 5 s^−1^. This value of Ks of our prepared modified electrodes is higher than Hb/graphene-Pt/GCE (0.14 s^−1^) [34]. This indicates that GNPs-GR-SDS composite is an excellent promoter for the electron transfer between Hb and electrode.

**Figure 6.**
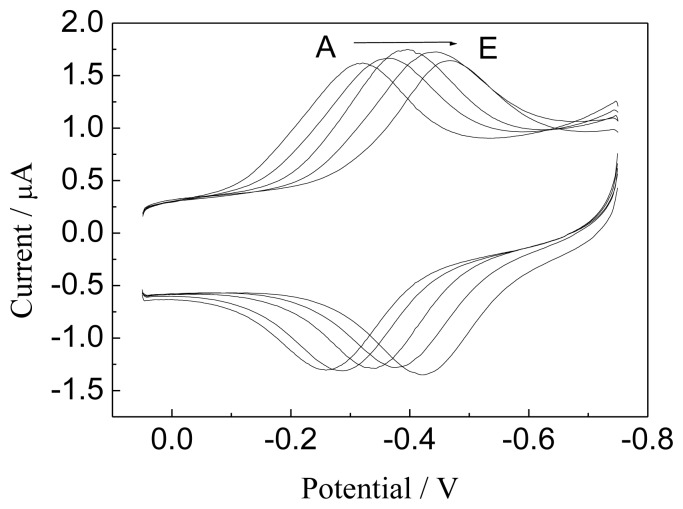
Cyclic voltammograms for Hb/GNPs-GR-SDS/BPG modified electrode at scan rate 0.1 V^−1^ in buffer solutions at different pH values. pH value(from A to E): 5, 6, 7, 8 and 9.

The effect of the pH of the supporting electrolyte on the peak potentials of the Hb was also investigated (Figure 6). Both reduction and oxidation peak potentials of the heme Fe(III)/Fe(II) redox couples of Hb are obviously dependent on the pH value in the range of 5.0 to 9.0. The apparent formal potentials of the Hb heme Fe(III)/Fe(II) redox couple, which were estimated as the midpoint ofcathodic and anodic peak potentials, had shifted linearly to the negative direction with the increase of pH value with a slope of -49.6 mV pH ^−1^, less than the theoretical value of -59 mV pH^−1^ at 25 °C for a reversible proton-coupled single electron transfer [35,36]. Although future research is needed to explore this interpretation, one thing for sure is that the electron transfer of Hb heme Fe(III)/Fe(II) couple in GNPs-GR-SDS films is accompanied by proton transport, which is also found for Hb-AQ films [37] and Hb-DDAB-clay [38] films:

$${\text{Hb heme Fe}(\text{III})+\text{H}}^{+}+{\text{e}}^{-}\to \text{Hb heme Fe}(\text{II})$$

### 3.3. Electrocatalytic Activity of Hb/GNPs-GR-SDS/BPG Electrode for the Reduction of Nitric Oxide

**Figure 7.**
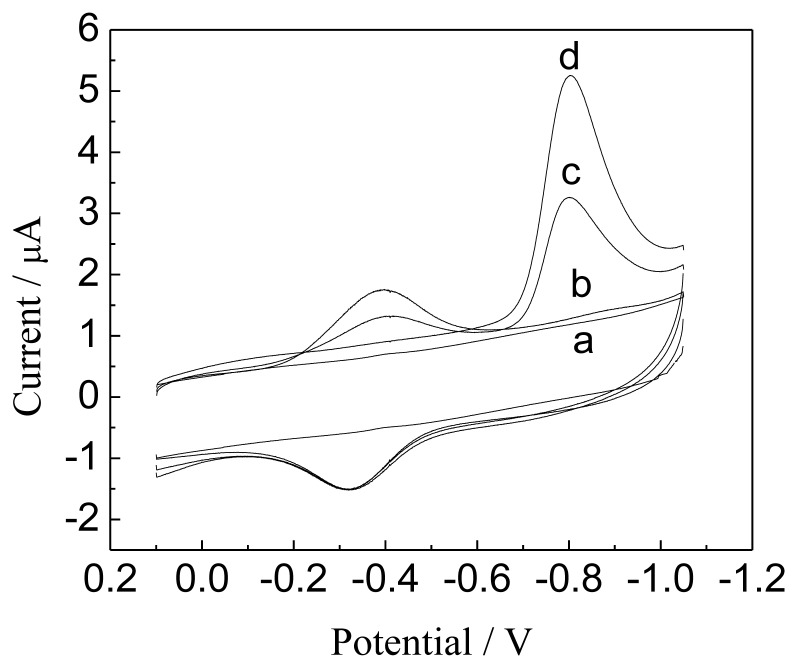
CVs of bare BPGE (**a**: 0.02 mM NO) and Hb/GNPs-GR-SDS/BPG modified electrode in PBS (pH 7.0) containing NO (**b**) 0, (**c**) 0.04 and (**d**) 0.08 mM. The scan rate is 100 mV·s^−1^.

The resulting Hb/GNPs-GR-SDS/BPG electrode exhibited a good electrocatalytic activity toward the reduction of NO. Figure 7 shows CVs of the Hb/GNPs-GR-SDS modified electrode and bare BPGelectrode in the absence or the presence of NO. With added NO, no peaks can be observed at a bare BPGE (Figure 7(a)). However, at the Hb/GNPs-GR-SDS modified electrode, an obvious new cathodic peak appeared at -0.82 V, which is due to the reduction of NO. Meanwhile, the cathodic peak current of the heme Fe(III)/Fe(II) redox couple decreased and its anodic peak current increased with increasing the concentration of NO (as shown in Figures 7 (b–d)), indicating a typical electrocatalytic reduction process.

**Figure 8.**
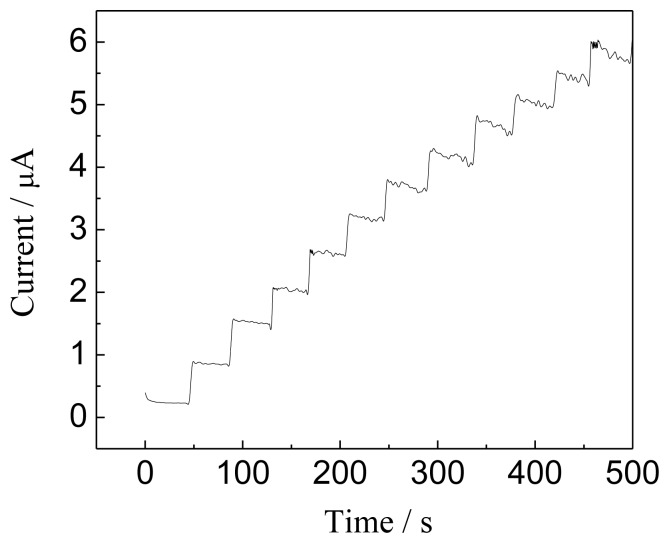
Amperometric response of Hb/GNPs-GR-SDS/BPG modified electrode to NO. Conditions: a -0.82 V constant potential modulated with 50 mV pulse in the time intervals of 0.5 s; successive additions of 2 μL of 3.6 mM NO to 10 mL of 0.1 M PBS, pH 7.0, and the stirring rate of solution is 400 rpm.

To further examine the response feature of the Hb/GNPs-GR-SDS electrode to NO, the amperometric response of the Hb/GNPs-GR-SDS modified BPG electrode to NO was recorded while successively adding NO to a continuous stirred PBS solution. In this process, a potential of −0.82 V was applied to the working electrode (Figure 8).

The reductive current increases to reach a stable plateau within 3 s when NO is added to the buffer solution. This suggested that the response of the electrode to NO should be a quick responsive process. The experimental results showed that the amperometric response current is linear when the concentration of NO is in the 0.72-7.92 μM range. In addition, the detection limit of the biosensor was evaluated to be 1.2 × 10^−8^ mol·L^−1^ based on 3 signal-noise ratio. The resulting response suggests that the Hb/GNPs-GR-SDS/BPG electrode can be used as a reagentless biosensor to detect NO. The determined parameters of this sensor and other modified electrodes used for the determination of NO are listed in Table 1. Compared with the existing reports [39-45], this method to prepare a Hb/GNPs-GR-SDS/BPG modified electrode with high sensitivity, wide linear range and good repeatability is convenient.

### 3.4. Stability and Reproducibility of the Biosensor

The stability of the Hb/GNPs-GR-SDS electrode was investigated. Even when 400 continuous cyclic scans were carried out in the potential range from 0.1 V to −1.0 V with a 100 mV/s scan rate, no obvious change of the CV curve can be observed, suggesting that proteins can be tightly immobilized on the surface-modified BPG electrode. Eight repetitive experiments were carried out with the buffer solution containing 0.72 μM NO. The relative standard derivation for eight repetitive measurements isbelow 2.3%. The Hb/GNPs-GR-SDS modified electrode that was left to stand in pH 7.0 PBS could keep its activity for at least two weeks.

## 4. Conclusions

In conclusion, a gold nanoparticle-graphene nanosheets suspension can be prepared though the chemical reduction method using SDS as protector and disperser. Hb adsorbed on the GNPs-GR-SDS modified electrode via the electrostatic interactions retains its native secondary protein structure and the direct electron transfer was achieved. The CVs of a Hb/GNPs-GR-SDS/BPG modified electrode exhibited a pair of redox peaks corresponding to the electroactive center of Hb and showed good catalytic activity for the reduction of NO. The developed approach can be potentially useful in the fields of catalysis, fuel cells, and other related fields.

## Figures and Tables

**Table 1.** Comparison of the proposed method with other electrochemical methods for determination of NO.

**Electrode**	**Linear Range (M)**	**Detection Limit (M)**	**Ks (s^−1^)**	**Reference**
Hb/cyanoethylcellulose-modified glassy carbon electrode	1.12 × 10^−6^∼4.72 × 10^−5^	2.0 × 10^−8^	—	[39]
poly[*N*-(2-methacryloyloxyethyl)pyrrolidone]-*block*-poly[glycidylmethacrylate]/Hb- modifiedglassy carbon electrode	4.5 × 10^−7^∼10.0 × 10^−6^	3.2 × 10^−7^	1.03 ± 0.05	[40]
cytochrome c/sodium dodecylsulfate/polyacrylamide-modified glassycarbon electrode	8.0 × 10^−7^∼9.5 × 10^−5^	1.0 × 10^−7^	1.56	[41]
Hb/gold colloids-modified carbon paste electrode	9.0 × 10^−7^∼3.0 × 10^−4^	1.0 × 10^−7^	3.72	[42]
Hb/poly(diallyldimethyl-ammoniumchloride)- functionalized graphenesheets/room temperature ionicliquid-modified glassy carbon electrode	2.0 × 10^−7^∼3.26 × 10^−5^	4.0 × 10^−8^	—	[43]
Hb/didodecyldimethylammonium bromide-modified powder microelectrode	1.90 × 10^−6^∼2.8 × 10^−5^	2.0 × 10^−7^	—	[44]
cytochrome c /polymerized ionic liquid-graphene/BPG electrode	1.05 × 10^−6^∼1.37 × 10^−5^	7.0 × 10^−7^	2.39	[45]
Hb/gold nanoparticles/graphene- modified basal plane graphite electrode	7.2 × 10^−7^∼7.92 × 10^−6^	1.2 × 10^−8^	48 ± 5	This work
